# Critical illness-related corticosteroid insufficiency (CIRCI) - an overview of pathogenesis, clinical presentation and management

**DOI:** 10.3389/fendo.2024.1473151

**Published:** 2024-11-06

**Authors:** Joanna Sobolewska, Lukasz Dzialach, Pawel Kuca, Przemyslaw Witek

**Affiliations:** Department of Internal Medicine, Endocrinology and Diabetes, Medical University of Warsaw, Warsaw, Poland

**Keywords:** critical illness-related corticosteroid insufficiency, CIRCI, intensive care, sepsis, septic shock

## Abstract

According to the Society of Critical Care Medicine, critical illness-related corticosteroid insufficiency (CIRCI) characterizes hypothalamic-adrenal axis insufficiency following acute medical conditions of various causes, i.e., sepsis, septic shock, acute respiratory distress syndrome, community-acquired pneumonia, and status after major surgical procedures. Due to highly variable etiology, understanding the pathomechanism and management of CIRCI assumes relevance for all centers providing intensive care. During CIRCI, multiple peripheral adaptations develop, and cortisol distribution volume increases due to hypothalamic-adrenal axis dysregulation, alterations in cortisol metabolism, and tissue resistance to corticosteroids. The proper diagnosis and treatment of CIRCI may be challenging in many cases. Although we have been acquainted with CIRCI since 2008, it remains a difficult condition with widely variable approaches among clinicians due to inconsistent high-quality study results determining the effect of corticosteroids on mortality. Corticosteroids are widely used in acutely ill patients, highlighting the necessity for reliable knowledge to support crucial clinicians’ decisions in daily medical practice. In this review, we provide an overview of the clinical management of patients with CIRCI based on current recommendations and selected studies.

## Introduction

Critical illness-related corticosteroid insufficiency (CIRCI) is a clinical condition with demanding diagnostics and individualized management. Since it may develop in the course of medical conditions of highly variable etiology, understanding the pathomechanism of CIRCI, as well as its manifestations, seems to be crucial for physicians of multiple specialties (1–3). Despite the numerous studies that have been presented, CIRCI remains a poorly understood clinical state, and the currently available knowledge still leaves many outstanding concerns unsolved. Our article aims to summarize current recommendations and selected research as on diagnostics and management of patients with CIRCI. The introduction of the term “CIRCI” is dated back to 2008, the year the Society of Critical Care Medicine (SCCM) used it to describe hypothalamus-pituitary-adrenal (HPA) axis insufficiency in the course of acute medical conditions (4). Previously, “relative adrenal insufficiency” was used (5). The significant focus of the CIRCI terminology has been to emphasize functional etiology, thus reflecting the concept that adrenal insufficiency (AI) in critical conditions may develop without structural defects in the HPA axis (1).

## The pathogenesis of CIRCI

The pathogenesis of CIRCI involves dysregulation of the HPA axis, alteration of cortisol metabolism and tissue resistance to corticosteroids (2, 6). The HPA axis response to systemic inflammation during critical conditions may be reduced or dysregulated, and the reactions previously considered adaptive may be inadequate in the acute condition (2). However, a prompt increase in systemic corticosteroid availability is then essential to efficiently prevent an inadequate immune response but also to induce cardiovascular (such as fluid retention, vasoconstriction) or metabolic effects by enhancing catabolism and decreasing anabolism (5, 7, 8). In acute conditions, multiple peripheral adaptations develop after brief activation of the HPA axis to maintain increased systemic availability of cortisol without its increased production (7).

Cortisol is secreted in a pulsatile pattern following a circadian rhythm, with physiological peak concentrations in the morning and a drop in concentration during the subsequent hours (2, 6, 9, 10). The HPA axis is crucial in maintaining homeostasis, and its proper activity is based on the principle of feedback, but in acute conditions, this regulation is far more complicated (2, 6, 7, 9, 10). Critical conditions of various etiologies, through neuronal and inflammatory signals, induce an accelerated release of adrenocorticotrophic hormone (ACTH) mediated by corticotropin-releasing hormone (CRH) and arginine vasopressin (AVP), resulting in a disruption of the circadian rhythm of cortisol production (2, 5, 7, 8). In this group of patients, hypercortisolemia most likely develops secondary to decreased cortisol metabolism rather than an increase in adrenal sensitivity to ACTH (2). Initially, cortisol concentrations rise in response to a significant increase in ACTH concentrations, which declines to near basal levels if inflammation persists for a prolonged duration (9). The response of the HPA axis to critical conditions is divided into an acute phase (a few minutes after the initial damage), a subacute phase (a few hours to several days after the initial damage) and a chronic phase (more than a few weeks after the initial damage) (5). The acute phase is characterized by a rapid increase in cortisol levels in reaction to an increase in ACTH levels and numerous peripheral adaptations (5). This relationship has been demonstrated in patients hospitalized in the Intensive Care Unit (ICU), where elevated ACTH levels occurred only transiently, such as during surgical procedures (7). Similarly, a study by Raff et al., indicated that patients with sepsis admitted to the ICU, evaluated within 24 hours, had elevated serum total and free cortisol levels but without an accompanying sustained increase in ACTH concentrations (11). However, this study had its limitations, as it involved a relatively small number of patients with sepsis (22 patients) (11). In a prospective study involving 392 critically ill patients requiring ICU hospitalization for more than seven days, ACTH levels were reduced or normal until the 28th day of hospitalization, and free plasma cortisol levels were elevated (12).

Cortisol is principally metabolized in the liver and kidneys, and the essential enzymes responsible for the initial stages of metabolism are 5 α/β-reductase and 11β-hydroxysteroid dehydrogenase type 2 (11β-HSD2), respectively, whose activity decreases in response to inflammation (2, 5). This prolongs the half-life of cortisol, thereby maintaining sufficiently increased systemic corticosteroid concentrations (7). Similarly, the inactive 11β-HSD2-mediated cortisol metabolite- cortisone, formed in the kidneys, may be converted back to cortisol in extra-adrenal tissues such as the liver, adipose tissue and muscles due to increased 11β-hydroxysteroid dehydrogenase type 1 (11β-HSD1) activity, which is modulated by inflammatory cytokines (2, 5, 13). The produced mediators, including TNF-α and IL-1β, affect the expression of 11β-HSD, which alters the sensitivity of cells to endogenous corticosteroids (13–15). Elevated pro-inflammatory cytokines during acute conditions can also inhibit adrenal cortisol synthesis and induce tissue resistance to corticosteroids (1). In a study by Boonen et al. involving 158 ICU patients and 64 controls, plasma total and free cortisol levels were higher in the experimental group, while ACTH levels were lower than in the control group (16). Moreover, the experimental group had significantly higher cortisol production and decreased cortisol clearance, resulting in a 3.5-fold increase in cortisolemia compared to the control group (16). In critically ill patients, hypercortisolemia is not followed by high ACTH concentrations, which has been described as “ACTH-cortisol dissociation”, emphasizing the role of the adrenal response in the pathomechanism of CIRCI (5, 11). In a study conducted by Boonen et al., it was shown that in the course of critical conditions, elevated cortisol levels were associated with suppressed nocturnal pulsatile ACTH secretion (17). This implies that hypercortisolemia in acute conditions develops by ACTH-independent mechanisms and may even result in negative feedback on the HPA axis (8, 11, 17). Hepatic cortisol metabolism may also be accelerated or prolonged by selected drugs used in acute conditions that modify the activity of cytochrome CYP3A4, such as amiodarone, macrolide group antibiotics (azithromycin, clarithromycin) or azole antifungals (1, 18). It has also been described that decreased cortisol metabolism may also occur secondary to critical illness-associated cholestatic liver dysfunction (3, 5, 19, 20).

**Figure 1:**
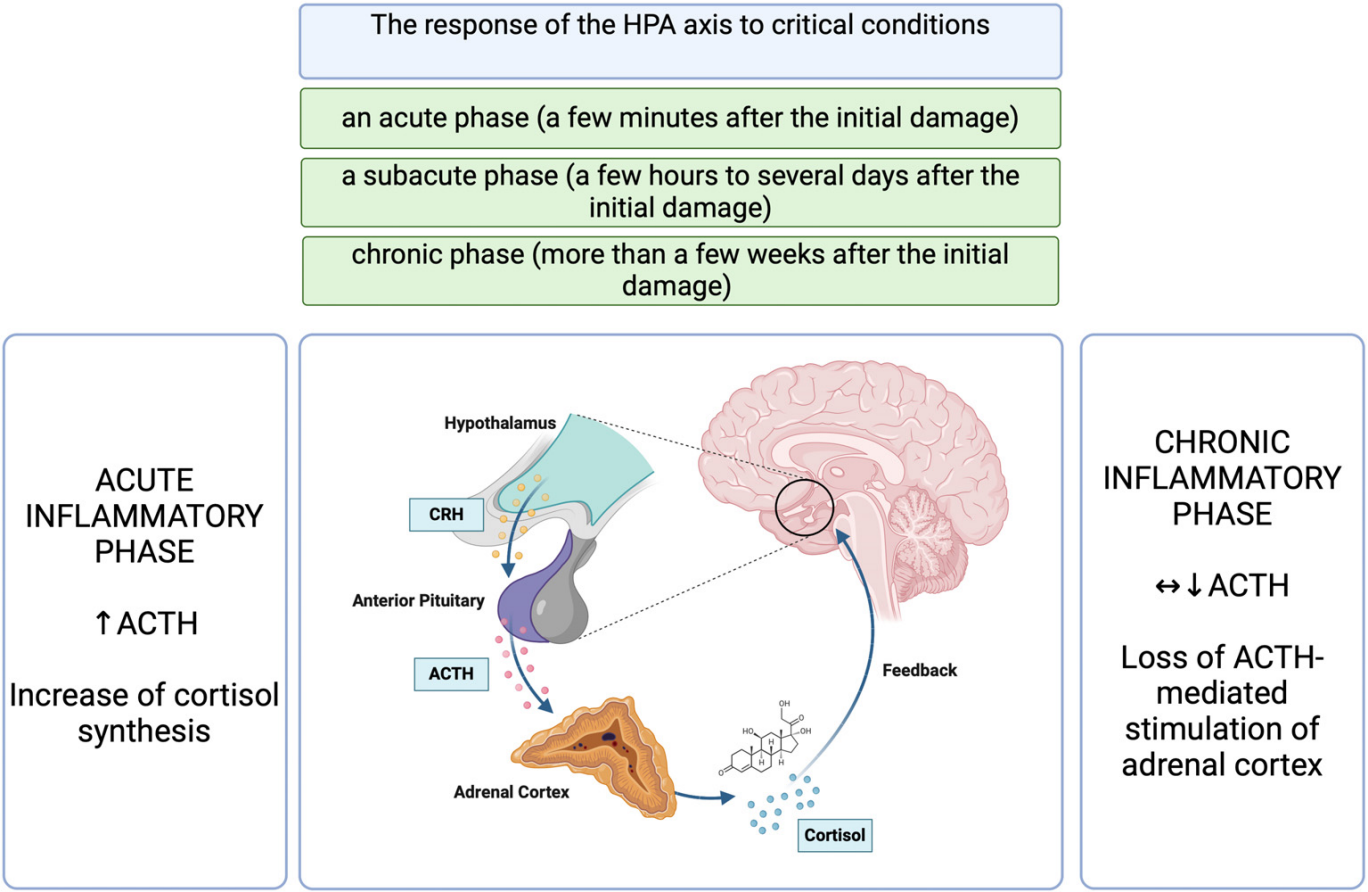
The response of the HPA axis to critical conditions (5, 7, 9, 11, 12). HPA, hypothalamic- pituitary- adrenal axis; CRH, corticotropin-releasing hormone; ACTH, adrenocorticotropic hormone. Figure created in BioRender.com.

**Figure 2:**
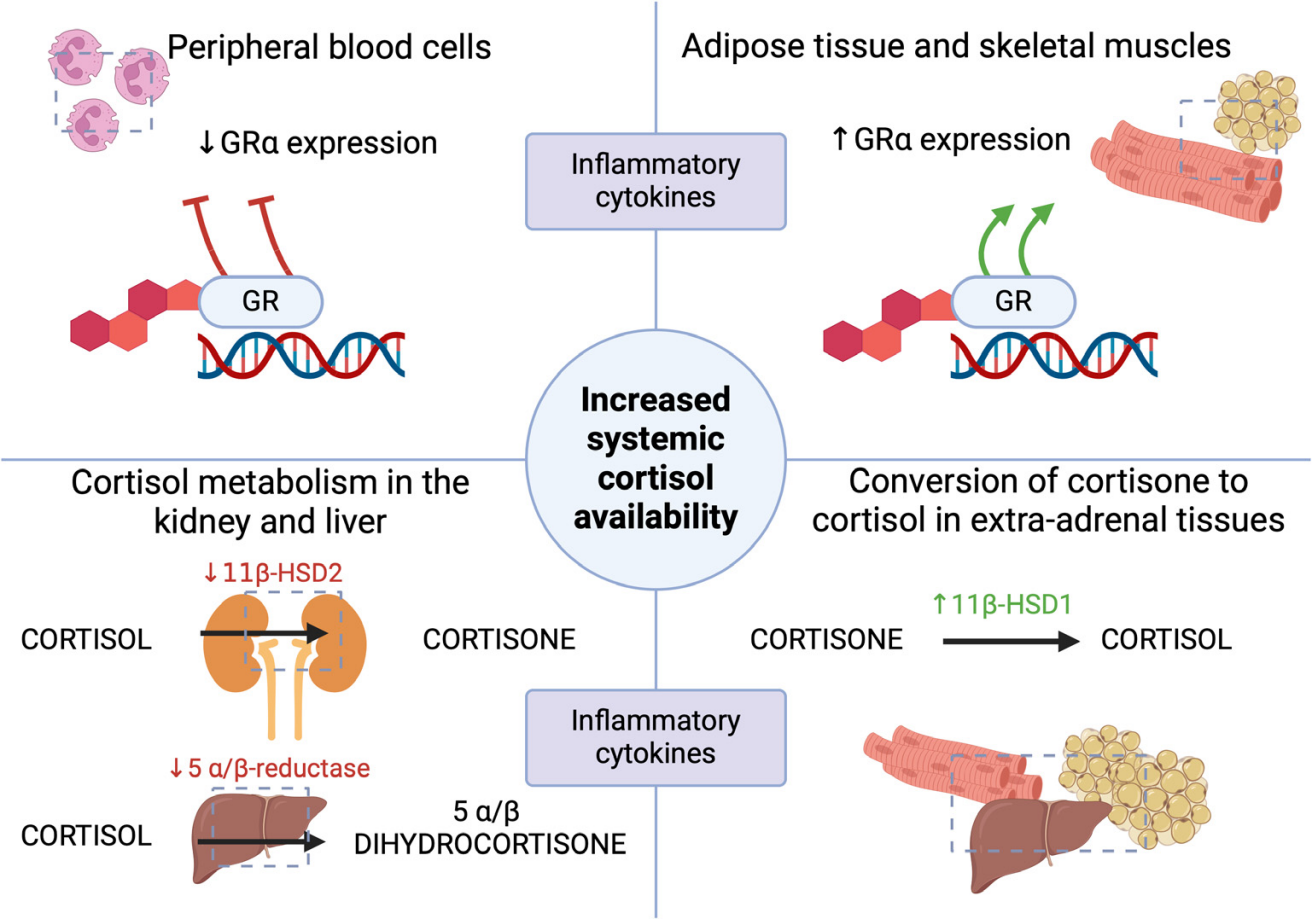
The mechanisms leading to the increased systemic availability of cortisol in acute conditions (1, 2, 5, 7, 9, 13–15, 22, 25). GR- glucocorticoid receptor; 11β-HSD1- 11β-hydroxysteroid dehydrogenase type 1; 11β-HSD2- 11β-hydroxysteroid dehydrogenase type 2. Figure created in BioRender.com.

The α glucocorticoid receptor (GR α) plays a crucial role in maintaining homeostasis and the physiological stress response (2). Both endo- and exogenous corticosteroids act through the GR (21). There are available studies in patients with sepsis that have shown reduced GR α expression in peripheral blood, which has been interpreted as a manifestation of generalized resistance to corticosteroids and, thus, a rationale for the use of hydrocortisone in higher doses to overcome resistance in sepsis or septic shock (7, 22). Sepsis is also characterized by an increased expression of the GR isoform β in circulating cells, resulting in an imbalance between GRα and GRβ (23, 24). However this insight’s limitation may be the differential effects of cortisol and synthetic corticosteroids depending on the target tissue (7). A study by Teblick et al. found that during critical illness, specific adaptations of GRα expression occur, and primarily neutrophils are responsible for the earlier observations of a decrease in GRα expression in peripheral blood cells of patients with sepsis (25). On the other hand, most further vital tissues and organs showed increased GRα action (25). This variation in GR expression could prevent immune- suppressive off-target effects of increased systemic cortisol availability (22). That observation also contradicts the generalized resistance to corticosteroids in acute conditions and the use of “stress” doses of hydrocortisone (25). However, some studies demonstrated that quantitatively adequate and prolonged glucocorticoid supplementation increased GRα number and function in both circulating and tissue cells, reversing critical illness-associated cellular glucocorticoid resistance (24, 26). When assessing the administration of glucocorticoid therapy, the current understanding of the role of activated GC-GRα in immunomodulation and the course of critical illness should be considered. **Figures 1**, **2** summarize the HPA axis’s response to critical conditions and adaptations leading to increased systemic cortisol availability.

In acutely ill patients, it is also worth considering other factors that may lead to iatrogenic adrenal suppression, such as the use of the antifungal drug, ketoconazole for opportunistic infections, or the anaesthetic agent etomidate (1, 3, 18), a side effect of which is becoming increasingly prevalent in life-threatening cases of hypercortisolemia (27, 28). Indeed, ketoconazole, one of the imidazole derivatives, is among the historical drugs used in managing Cushing’s disease because it inhibits cortisol synthesis by affecting 11β-hydroxylase activity (29, 30). Similar effects on the steroid synthesis pathway are also reported with etomidate, which additionally inhibits 17α-hydroxylase activity (29, 30). Further clinical conditions that may lead to iatrogenic adrenal suppression also include cases of adrenal hemorrhage, which can occur in septic patients with coagulopathies (1), assembling imaging methods such as ultrasound, computer tomography or magnetic resonance imaging valuable in such cases (3).

## The clinical manifestation of CIRCI

**Figure 3:**
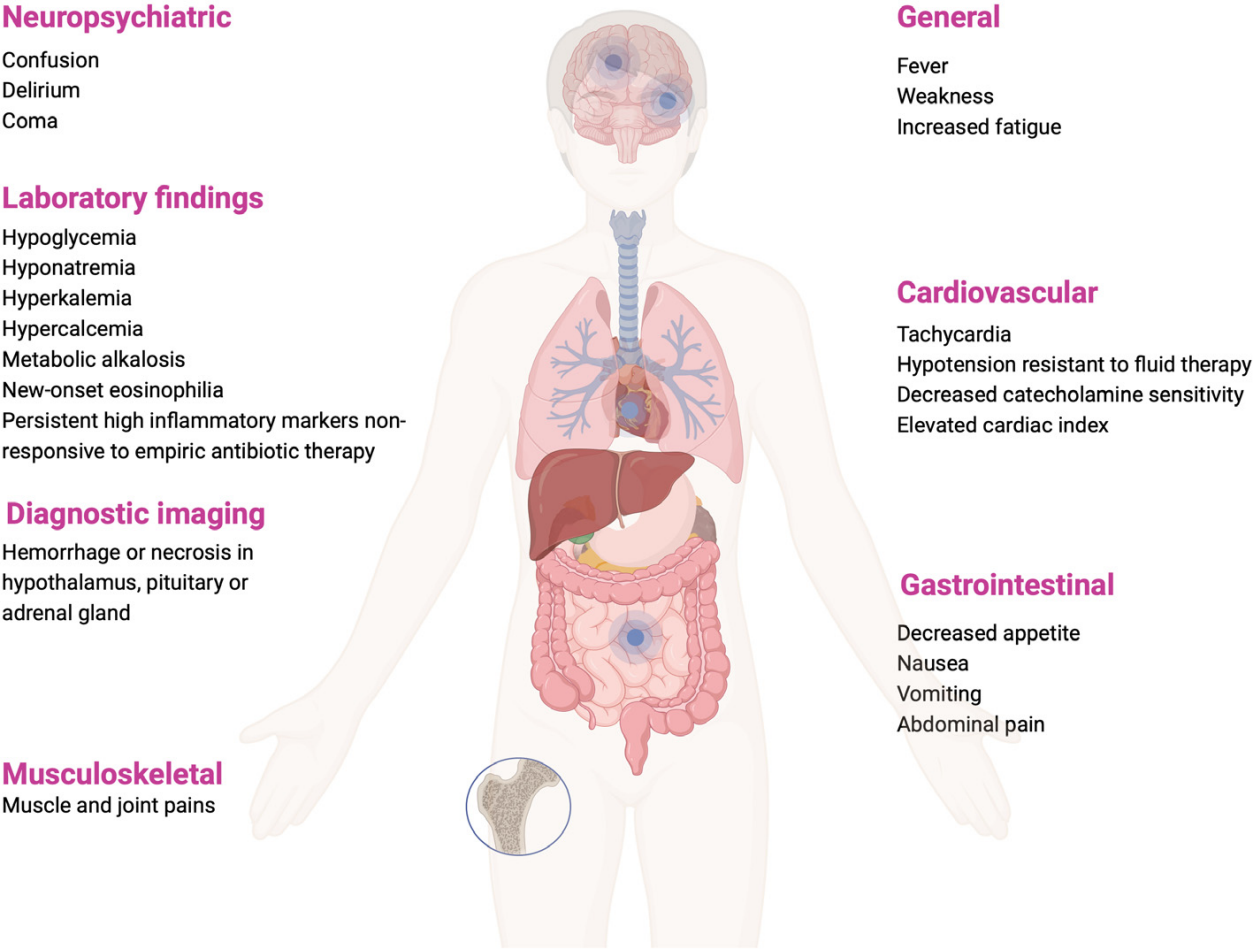
Clinical manifestations, laboratory and imaging findings that may occur in CIRCI (1, 3, 36). Figure created in BioRender.com.

CIRCI may develop in the course of sepsis, septic shock, acute respiratory distress syndrome (ARDS), community-acquired pneumonia, cardiac arrest, trauma, burns, and after extensive surgical procedures (2). Clinical manifestations range from nonspecific, such as fever and weakness, nausea and vomiting, to symptoms involving multiple systems, such as confusion, delirium, coma; hypoxia, or hypotension refractory to fluid resuscitation (1–3). Possible clinical symptoms and laboratory and imaging abnormalities during CIRCI are summarized on **Figure 3**. Electrolyte abnormalities found in the course of CIRCI, such as hyponatremia and hyperkalemia, may be masked by intensive fluid therapy, but hypoglycemia or eosinophilia should suggest the suspicion of CIRCI, as these abnormalities are relatively rare in acute conditions (1).

## Epidemiology of CIRCI

In a study by Hashemi-Madani et al. evaluating 99 patients admitted to the ICU, AI was found in 25.3% of patients, with no significant differences in the incidence of AI in patients with sepsis, severe sepsis or septic shock (31). Patients with positive blood cultures and greater C-reactive protein levels had a higher risk of developing AI; additionally, there was no significant difference in the average age of acutely ill patients with AI and those who did not develop hypocortisolism (31). In a retrospective single-center study assessing 145 patients with COVID-19 in critical conditions, 22.9% of these patients were likely to develop CIRCI (32). However, the results are slightly concerning, as in this group of patients, those who were treated with corticosteroids had a longer duration of mechanical ventilation, a higher risk of morbidity and mortality, and more severe organ dysfunction, emphasizing the unusual clinical manifestation of CIRCI in COVID-19 patients and appearing to be a factor for increased mortality in this group of patients (32). Similarly, in a study by Li et al., early (≤3 days after ICU admission) initiation of corticosteroid treatment (methylprednisolone was mainly administered) in patients with COVID-19 led to an increase in 90-day mortality (33).

Another important group are oncology patients with sepsis, who represent a high-risk subgroup for CIRCI, which may be overlapped by metastatic adrenal lesions or HPA axis dysfunction after radiation therapy (34). Existing data on the incidence of CIRCI in this group of patients appear to be limited, as diagnosed malignancy has formed an exclusion criterion in many trials (34). For instance, in a study by Sprung et al., oncological patients accounted for only 16.8% of the patients included (35). In a research by Bruno et al. evaluating 86 oncology patients with severe sepsis or septic shock, 59% of patients developed CIRCI, but the mortality rate in this study did not differ from the group that did not develop CIRCI (34).

## CIRCI diagnostics

The 2008 SCCM guidelines already recommended that random plasma or serum total cortisol measurement <10μg/dL or change in baseline cortisol <9 μg/dL (cortisol) at 60 min in 250 μg cosyntropin test be used to diagnose CIRCI (4). The 2017 SCCM guidelines do not indicate the superiority of either test in this indication (36). Endocrine Society recommends that the 250 μg corticotropin stimulation test is the gold standard for diagnosing primary AI (37). The term CIRCI does not include HPA axis abnormalities that were present before the onset of the acute condition (3). Even if the initial test results excluded CIRCI, it can develop in the subsequent days of infection, so it is essential to actively search for signs of AI and retest, especially if the patient’s general condition worsens (1). In the reviewed literature, data about using a test with 1 μg cosyntropin, i.e. low-dose ACTH test for the diagnosis of CIRCI also appear (1, 38–41), however, current SCCM guidelines do not recommend this test (36). It is noteworthy to mention here the results of a single-center study conducted by Marik et al. involving 59 patients with septic shock, in which, depending on the type of test performed, a varying percentage of patients met diagnostic criteria for AI - 22% of patients in the test with 1 μg and 8% of patients in the test with 250 μg corticotropin (38). In another study by Widmer et al., moderate and major stress situations were associated with higher peak cortisol concentrations after stimulation with 250 μg than after 1 μg cosyntropin (42). In a survey involving 189 patients with septic shock, Annane et al. divided patients into three groups according to the response obtained in the 250 μg cosyntropin test and baseline cortisol levels, identifying patients with basal cortisol >34 μg/dL and response in the cosyntropin test (cortisol ≤9 μg/dL) as those with the highest risk of death and a median survival time of five days (43). This study underscores that a low increase in cortisol concentration in a test with 250 μg cosyntropin is predictive of mortality, independently of baseline serum cortisol concentration (43). The previously mentioned adaptive modifications to increase the volume of cortisol distribution significantly affect the perception of the stimulation test with cosyntropin in critical conditions, as the incremental response of total cortisol concentration is reduced and free cortisol concentration is normal (3, 7). In proportion to the severity of the general condition, the level of cortisol binding globulin (CBG) decreases, as we discuss below, the volume of corticosteroid distribution rises, and the incremental concentration of total cortisol in the test with cosyntropin is more inhibited (3, 7). This emphasizes not considering the cosyntropin stimulation test as a tool to identify patients who should be treated with exogenous glucocorticoids (7). The SCCM recommendations also do not suggest evaluating the hemodynamic response to hydrocortisone in the diagnosis of CIRCI, rather than performing a test with 250 μg corticotropin, as well as an isolated determination of ACTH levels, which, as we have discussed, can vary in the course of acute conditions (36).

In patients with suspected CIRCI, it is not recommended to measure free plasma cortisol concentrations instead of total serum cortisol levels (36). An estimated 80-90% of circulating cortisol is bound to CBG, 10-15% to albumin, and the remaining percentage occurs in unbound form (1, 2, 5, 44). Both CBG and albumin are among the negative acute-phase proteins (2), and decline in critical conditions, and thus changes in CBG concentrations significantly complicate reliable assessment of free cortisol levels (1, 2, 5, 36). Their concentrations also decrease secondary to bleeding, enteropathy or dilution secondary to fluid resuscitation (5).

It is also noteworthy to outline the variability of cortisol’s binding capacity to CBG and albumin in relation to cortisol concentrations. CBG is a 50-60 kDa glycoprotein with a high affinity for cortisol (44, 45). An increase in cortisol secretion in response to psychological and physical stressors or tissue damage is part of the “fight or flight” reaction, and similarly, in critically ill patients, the severity of surgery or infection positively correlates with the degree of cortisolemia (1, 5, 46). CBG binding capacity is saturated at cortisol concentrations in the range of 22-25 μg/dL, while when concentrations are higher, the proportion of albumin-bound cortisol and free cortisol rises, and the fraction bound to CBG remains unaltered (2). CBG concentration, total plasma cortisol concentration and albuminemia, may also be used to estimate the amount of free cortisol in plasma, using Coolens’ formula in a modified version (5). However, this method should be interpreted cautiously in acutely ill patients (47). A study by Chan et al. highlighted the role of glycosylation as crucial in maintaining CBG function, both as a carrier of corticosteroids and as a modulator allowing differentiated release into tissues (44). Indeed, it was shown that the glycosylated form of CBG binds cortisol with significantly higher affinity than the non-glycosylated form and, interestingly, that an increase in body temperature by every two Celsius degrees doubles the concentration of free cortisol (44).

An interesting peripheral adaptation in acute conditions is also the increase in the distribution volume of cortisol due to decreased hepatic synthesis of cortisol-binding proteins and the altered affinity of cortisol for binding proteins in proportion to the severity of the disease (7). Recent findings suggest that the role of CBG during inflammation and sepsis is considerably increasing as a transporter protein delivering cortisol with immunomodulatory effects. Through a complex regulatory mechanism, in response to temperature and acidity, cortisol is directed to the highest-demand areas in sepsis conditions (45). The increase in free cortisol in acute conditions is also led by the effect of neutrophil elastase, which changes CBG to a form with low binding capacity (1, 5, 13, 45), as well as inflammatory cytokines that inhibit hepatic CBG synthesis (45). Decreased CBG levels affected one-third of patients with septic shock admitted to the ICU and were a risk factor for increased mortality, opening promising perspectives for rapid CBG assays as those with high prognostic value in sepsis (45). In a study by Dubey and Boujoukos, 39% of critically ill patients with concomitant decreased albumin levels had abnormal total serum cortisol levels despite preserved normal adrenal function (48). Although even if the free cortisol concentration could be measured reliably, it is difficult to estimate its recommended concentration, as it will differ depending on the severity of the patient’s general condition (1, 7). Similarly, salivary cortisol determination is not recommended because of the possible changes in free cortisol concentrations described above but also due to the lack of cooperation enforced by the patient’s critical condition, i.e., the inability of an unconscious or an intubated patient to use a salivette, and the limited availability of this type of assessment in many centers (36). Publications are available that evaluate free cortisol concentrations in saliva sampled in the morning (49), including after stimulation with synthetic ACTH (50),, proving the usability of these determinations in diagnosing AI (49, 50); SCCM guidelines, however, do not recommend the use of this assay (36). According to the „Consensus on diagnosis and management of Cushing’s disease”, the determination of free salivary cortisol, however, performed in the evening (late night salivary cortisol) is used in the diagnosis of Cushing Syndrome and the detection of disruption of the circadian nadir of cortisol secretion in this group of patients (51).

## Treatment with corticosteroids in selected clinical indications

Corticosteroids are likely among the most frequently used drugs in medicine (18). No conclusive data are available on the recommended dose, time of initiation and duration of therapy with corticosteroids in acute conditions, which, in clinical practice, have been used in patients with severe infections since the mid-20th century (21, 52). Synthetic corticosteroids bind glucocorticoid receptors with higher affinity, while mineralocorticoid receptors bind with lower affinity than endogenous corticosteroids (13). Furthermore, synthetic corticosteroids are not bound by CBG and are not inactivated by 11β-HSD, and thus exhibit stronger immunoregulatory potency than endogenous corticosteroids (13, 21). pa

Among clinicians, there are variable opinions about the usage of corticosteroids and their effect on survival in patients with sepsis (52). Sepsis and septic shock result in death from one in six to one in three patients (53). To properly expand on the issue, it is worth repeating the definition of sepsis and septic shock proposed by the International Consensus Sepsis- 3 (54). Sepsis is life-threatening organ dysfunction in the course of a dysregulated response to infection, as manifested as an increase of ≥2 points on the Sequential [Sepsis-related] Organ Failure Assessment (SOFA) scale (54). Septic shock refers to patients who require vasopressor therapy to ensure mean arterial pressure (MAP) ≥65 mmHg and serum lactate levels >2 mmol/L (>18 mg/dL) in the absence of hypovolemia (54). Septic shock is defined as a subgroup of sepsis presenting with more severe cellular, metabolic and hemodynamic abnormalities than in sepsis (54). Corticosteroids used in septic shock may accelerate the reversal of shock but may also have adverse effects on the adaptive capacity of the HPA axis (7). Sepsis is associated with an in-hospital mortality rate higher than 10%, while septic shock above 40% (54). The SCCM suggests that corticosteroids should not be used in adult patients with sepsis without associated septic shock (36). In the HYPRESS trial, with 380 adult patients with severe sepsis, evaluating whether hydrocortisone administration (200 mg in continuous intravenous infusion) prevents the development of septic shock, no significant differences were observed between the hydrocortisone-treated and placebo-treated groups (21.2% vs 22.9%) (55). In addition, in the hydrocortisone-treated group, patients were more likely to develop secondary infections and hyperglycemia (55).

The Surviving Sepsis Campaign’s 2021 recommendations advise the usage of intravenous corticosteroids in adult patients with septic shock (weak recommendation; moderate quality of evidence), especially intravenous hydrocortisone at a dose of 200 mg/day in fractionated doses every six hours or as a continuous infusion (53). Considering the drug’s pharmacokinetics, the use of instant-release hydrocortisone, when dosed two/three times a day, does not simulate the physiological circadian rhythm of cortisol secretion, including its natural, gradual decrease during the day (18). Hence, intravenous infusion of hydrocortisone with the potential ability of flow rate modification has an advantage, improving the pharmacokinetic profile, which is possible in critically ill patients but impossible in patients chronically substituted with hydrocortisone on an outpatient basis (18).

The treatment of hydrocortisone at a dose of 50 mg every six hours, leads to supra-physiological cortisol concentrations, which may be important for the underlying tissue resistance to corticosteroids in CIRCI (1). The daily doses of hydrocortisone (200-300 mg) recommended for treating septic shock are equivalent to at least ten times the daily replacement dose for healthy individuals and about four times the average daily cortisol production in critically ill patients (16). It is suggested to initiate corticosteroid treatment when using doses of norepinephrine or epinephrine ≥ 0.25 µg/kg/min at least four hours after the start of treatment to maintain the recommended MAP (53). In clinical practice, hydrocortisone is also used in patients in septic shock when there is an increasing need for pressure amines in the absence of accompanying AI (3).

It is worth presenting three key randomized trials, which were the ones that significantly varied clinicians’ opinions. In the ADRENAL trial, which enrolled patients treated with vasopressors and inotropic drugs for ≥4 hours to maintain MAP >60 mmHg, hydrocortisone treatment was given for a maximum of seven days or shorter until discharge from the ICU or death (56). The hydrocortisone treatment used in this study did not improve the 90-day survival of patients with septic shock compared to the placebo group (56). The hydrocortisone-treated group had a more rapid resolution of shock and less frequent requirement for blood transfusion compared to the placebo-treated group (56). In the multicenter, double-blind, randomized APROCCHSS trial, among 1,241 patients enrolled in the study, 90-day mortality from any cause was 6% lower in the group of patients treated with hydrocortisone along with fludrocortisone than in the placebo group (43% vs 49%, respectively) (52). Adding fludrocortisone (50 μg orally) to treatment was supposed to provide additional mineralocorticoid potency (52). Similarly, the number of days without the requirement for vasopressors and those without organ failure were higher in the hydrocortisone plus fludrocortisone group compared to the placebo group (52). In contrast, the number of days without mechanical ventilation was similar in both groups and importantly, hyperglycemia was more frequent in the group of patients treated with hydrocortisone plus fludrocortisone (52). The risk of secondary infections, gastrointestinal bleeding and neurological consequences was not significantly higher in the hydrocortisone plus fludrocortisone treatment group than in the placebo group (52). An interesting perspective on the role of hyperrenin hypoaldosteronism in critical conditions was presented by Nethathe et al., where the term critical illness-related mineralocorticoid insufficiency (CIRMI) was proposed for this type of dysfunction and indicated that hydrocortisone and fludrocortisone combination therapy should be considered in patients with septic shock (57). Another study that is also worth mentioning in the context of the role of mineralocorticosteroids is the FluDReSS trial, designed to evaluate hydrocortisone and fludrocortisone combination therapy in critically ill patients, which has completed recruitment and the results of which we are currently awaiting (58). In the CORTICUS trial, which evaluated the efficacy and safety of low-dose hydrocortisone therapy among patients with septic shock, treatment did not improve survival (35). Hydrocortisone treatment did not significantly affect shock reversal, but shock resolved more promptly in the hydrocortisone-treated group than in the placebo group (35). This group also experienced more superinfections (35). The studies presented are summarized in **Table 1**. They appear to provide contrasting results, which may be explained by the fact that the patients in the CORTICUS and ADRENAL trials were less sick than those in the APROCCHSS trial, considering, for example, the higher vasopressors required. In addition, in both the ADRENAL and APROCCHSS studies, hydrocortisone was administered for seven days, but the pattern of drug administration differed-fractional doses versus continuous infusion. Controversy has arisen over the differing results of the indicated studies, enough that Annane, in his article “Why My Steroid Trials in Septic Shock Were ‘Positive’?” accessibly summarized the five studies, indicating, among other reasons, that patients in worse general condition, i.e., with higher vasopressor requirements and greater severity of organ failure, benefited significantly from corticosteroid treatment (59). Results are also available from other studies that varied in the number of patients who experienced a reduction in the duration of septic shock during corticosteroid treatment (60, 61).

**Table 1:** ADRENAL (56), APROCCHSS (52) and CORTICUS (35) trials overview.

Study design	Trial		
	ADRENAL (56)	APROCCHSS (52)	CORTICUS (35)
Corticosteroid used	hydrocortisone	hydrocortisone combined with fludrocortisone	hydrocortisone
Route of drug administration	intravenous infusion	intravenous bolus (hydrocortisone); orally (fludrocortisone)	intravenous bolus
Dose per day	200 mg	200 mg (50mg every 6 hours) [hydrocortisone] 50 μg (fludrocortisone)	200 mg (50mg every 6 hours)
Treatment duration (days)	7	7	5
Number of patients	3658	1241	499
Mean age in the corticosteroid group (years)	62.3 ±14.9	66.0 ± 14	63.0 ± 14
Primary outcome corticosteroid vs placebo group* (%)	27.9 vs 28.8 P=0.50	43.0 vs 49.1 P=0.03	34.3 vs 31.5 P=0.51

*The primary endpoint in the ADRENAL study and APROCCHSS was 90-day mortality, in the CORTICUS study 28-day mortality (35, 52, 56).

The SCCM recommends the use of hydrocortisone <400 mg intravenously or hydrocortisone equivalent in patients with severe forms of community-acquired pneumonia, indicating that corticosteroids shortened the length of hospitalization, reduced the possibility of the requirement for mechanical ventilation, the development of ARDS, but increased the risk of hyperglycemia, without causing other clinically significant complications (62).

Another important concern in a group of such interdisciplinary patients is cardiogenic shock, which has similar hemodynamic, inflammatory patterns and complications as septic shock and may involve CIRCI (63). In a single-center study conducted by Ducroq et al. involving 79 patients with cardiogenic shock, 42% of patients developed CIRCI, but in this research, this was not associated with an impact on 90-day mortality (63). Notably, exclusion criteria in this study included current ongoing corticosteroid therapy and use of etomidate (63).

Exogenous corticosteroid therapy has limitations, ranging from immunosuppressive effects to adrenal cortex inhibition (1, 13, 18). Corticosteroid-treated patients have been found to experience a significantly increased risk of gastrointestinal bleeding and perforation during hospital treatment, which was not found in outpatients (64). Through suppression of ACTH production, adrenal atrophy may occur, which, depending on the length of corticosteroid treatment, can persist even months after its termination (1). The possibility of developing the adrenal suppression described should be considered on an individualized basis for the patient, especially when corticosteroid therapy has lasted more than three weeks, and the patient has received more than 30 mg of hydrocortisone daily (or 7.5 mg prednisolone/day; or 0.75 mg dexamethasone/day) (1). Frequently used in medical nomenclature, “high” and “low” doses of corticosteroids vary depending on the indication for which they are applied (18). In rheumatological indications, the “very high dose” is referred to when the administration exceeds 100 mg of prednisone per day; in the indication of acute respiratory failure, the use of >30 mg of prednisone daily is similarly referred to as “high dose” (18). In a study by Min et al. evaluating the effect of short-term (1-2 weeks) use of high-dose corticosteroids (methylprednisolone at a daily dose of 48mg, equivalent to about 60 mg of prednisolone), in hospital but also outpatient pharmacotherapy, adverse effects were observed in 1/3 of patients (65). Once CIRCI is diagnosed and hydrocortisone treatment is initiated, therapy should be conducted with the minimum effective dose, and discontinuation should be attempted after recovery (3). The most recent SCCM guidelines do not specify how to conduct corticosteroid treatment discontinuation in CIRCI (36), but current Surviving Sepsis Campaign recommendations indicate that corticosteroids should be administered when vasopressor requirement persists (53). Interestingly, a retrospective cohort study by Carabetta et al. evaluated the impact of abrupt and gradual withdrawal of corticosteroids in patients in septic shock (66). Patients in the gradually reduced-dose group were more likely to experience hemodynamic instability than those in the abrupt dose reduction group (21.9% vs. 10.7%, respectively) (66). Hyperglycemia within 24 hours of the last dose of hydrocortisone was more frequent in the abrupt dose reduction group, while length of ICU hospitalization and in-hospital mortality were similar in both groups (66). In another retrospective study by Sobolewski et al., similarly, hemodynamic instability was more frequent in the gradual withdrawal group than in the abrupt discontinuation group (17.1% vs. 2.2%), while worsened glycemic control occurred in the gradual withdrawal group (67). Vanhorebeek et al., evaluated whether neuroendocrine disorders involving, among others, the corticotropic axis found during ICU hospitalization would resolve after discharge and how the function of this axis would fare in the evaluation made at five years following the acute condition (68). Total and free cortisol, as well as CBG levels, were similar in the study and control groups, while residual thyrotropic axis abnormalities persisted (68).

## Conclusions

The data presented here demonstrate the lack of comprehensive research on CIRCI, which results in highly variable current approaches among clinicians. Diagnosing CIRCI in critically ill patients and managing corticosteroid therapy may be demanding due to limited data from high-quality clinical trials, particularly regarding the duration of corticosteroid treatment. Despite shedding new light on corticosteroid usage, numerous aspects of CIRCI still require further clarification and research. That underscores the importance for clinicians to expand their knowledge in this area of intensive care medicine and emphasizes the essential need for accurate patient assessment in critical conditions of various etiologies, particularly when the patient does not respond or worsens during ongoing therapy.
